# Limited cryoablation reduces hospital stay and opioid consumption compared to thoracic epidural analgesia after minimally invasive repair of pectus excavatum

**DOI:** 10.1097/MD.0000000000029773

**Published:** 2022-08-05

**Authors:** Seung Hwan Song, Duk Hwan Moon, Yon Hee Shim, Hyunjoo Jung, Sungsoo Lee

**Affiliations:** a Department of Thoracic and Cardiovascular Surgery, Sanggye Paik Hospital, Inje University College of Medicine, Seoul, Republic of Korea; b Department of Thoracic Surgery, Gangnam Severance Hospital, Yonsei University College of Medicine, Seoul, Republic of Korea; c Department of Anesthesiology and Pain Medicine, Gangnam Severance Hospital, Yonsei University College of Medicine, Seoul, Republic of Korea.

## Abstract

pain following minimally invasive repair of pectus excavatum (MIRPE) is a critical concern that leads to a prolonged hospital stay and high doses of opiates administered to the patients. This study aimed to evaluate the efficacy of intraoperative cryoanalgesia (cryoablation of the intercostal nerves) during MIRPE. We retrospectively analyzed the data of 64 patients who underwent MIRPE and received cryoanalgesia or epidural analgesia between January 2019 and January 2021. The oral morphine milligram equivalent (MME) was used to calculate the dosage of opioid agents. The median age was 15 years (range, 4–33 years). The median postoperative hospital stay was 4 days (range, 2–6 days), with a median oral MME consumption of 45 mg (ranging from 0 to 1360 mg). Cryoanalgesia was performed in 38 patients, and epidural analgesia was administered to the remaining 26 patients. The cryoanalgesia group had a significantly lesser pain score, shorter postoperative hospital stay and lower oral MME consumption than the epidural analgesia group (5 vs 2; *P* < .001, 3 days vs 5 days; *P* < .001, 19 mg vs 634 mg; *P* < .001). Cryoanalgesia appears to reduce postoperative hospital stay and opioid consumption compared with epidural analgesia. The outcomes of this study indicate that cryoanalgesia might be a safe and effective method for pain control following MIRPE.

## 1. Introduction

Pectus excavatum, an inward displacement of the sternum, is the most common congenital chest wall deformity, with a prevalence of approximately 1 in 400 to 1 in 1000 live births.^[1]^ The Nuss procedure using a substernal bar, a minimally invasive repair of the pectus excavatum (MIRPE), is the most commonly performed surgical procedure for correction.^[2,3]^ Compared with the Ravitch procedure, MIRPE allows for smaller incisions, shorter duration of surgery, and less blood loss, with excellent postoperative outcomes.^[4,5]^ Despite these benefits, postoperative pain is a major disadvantage of MIRPE.

Following MIRPE, pain management is an important factor in determining the postoperative course. Epidural analgesia and intravenous patient-controlled analgesia are widely used to manage postoperative pain.^[6]^ However, these techniques are useful within the first 2 to 3 postoperative days and can cause severe problems, such as respiratory depression.^[6,7]^ Hence, cryoanalgesia has recently been adopted and employed to overcome these drawbacks.

Cryoablation of the intercostal nerves, also known as cryoanalgesia during MIRPE, has been reported to effectively and to safely control postoperative pain.^[8,9]^ However, we believe that cryoanalgesia in the previous studies was performed at more levels of the intercostal nerves than necessary. In these studies, cryoanalgesia was performed at 5 levels on each side: the level of bar insertion and 2 interspaces above and 2 below. In this study, we evaluated whether intraoperative cryoanalgesia could be applied at fewer levels of the intercostal nerves (the level of bar insertion and one interspace above and below) and still provide effective control of postoperative pain in patients with pectus excavatum.

## 2. Methods

### 2.1. Retrospective review and data collection

A retrospective chart review of the data of all patients who underwent MIRPE between January 2019 and January 2021 was performed. Patient data, such as demographics, symptoms, details of surgery, and type of analgesia (cryoanalgesia or epidural analgesia), were collected for review. In addition, the patients’ medical records of the postoperative course, including daily pain score, postoperative hospital stay, and pain medication used, were also reviewed. The opioid usage during the hospital stay was converted to oral morphine milligram equivalent (MME) for each patient. The study was conducted in accordance with the Declaration of Helsinki (revised in 2013) and approved by the local institutional review board (No.: 3-2020-0333).

### 2.2. Definitions

Postoperative hospital stay was defined as the number of days from the day of surgery to the day of discharge. Haller index was calculated by dividing the maximal transverse diameter of the thoracic cavity by the maximal anteroposterior diameter of the thoracic cavity on computed tomography scan. Complications were defined as any adverse events during or after the surgery, such as infection, bleeding, pneumothorax, hemothorax, bar displacement, conversion to sternotomy or thoracotomy, cardiopulmonary injury, pneumonia, reoperation, prolonged pain, readmission, or death.

### 2.3. General anesthesia

Atropine or glycopyrrolate was administered intravenously as premedication. When patients arrived at the operating room, standardized monitoring was initiated. Anesthesia was then induced with propofol 2 mg kg^-1^ followed by rocuronium 0.6 mg kg^-1^ for muscle relaxation to facilitate tracheal intubation. Anesthesia was maintained by administration of sevoflurane 1.5 to 2.5% and remifentanil 0.05 to 0.2 μg kg^-1^ min^-1^. About 20 minutes before the end of surgery, ondansetron or ramosetron were administered. At the end of surgery, any residual neuromuscular blockade was reversed with sugammadex or neostigmine.

### 2.4. Surgical technique

Sixty-four patients underwent MIRPE, which was performed by an experienced surgeon. MIRPE was performed using a double compression and complete fixation bar system.^[10]^ After general anesthesia and intubation, minimal bilateral incisions were made in the mid-axillary line. Two bent bars (lower and upper bars) were prepared. The lower bar was inserted to elevate the sternum, and the upper bar was inserted into the subcutaneous tissue, just above the sternum, using the same incisions. Two bolts were used to fix the upper and lower bars on both the left and right sides. The skin edge was pulled over the end of the bars to place the bars under the subcutaneous layer.

### 2.5. Patient selection

Before the introduction of the cryoablation machine in our department, postoperative pain after MIRPE used to be conventionally managed using epidural analgesia. Cryoanalgesia is being used to manage postoperative pain following MIRPE since the installation of the cryoablation machine. The patients in this study were selected sequentially.

### 2.6. Cryoanalgesia

**Figure 1.**
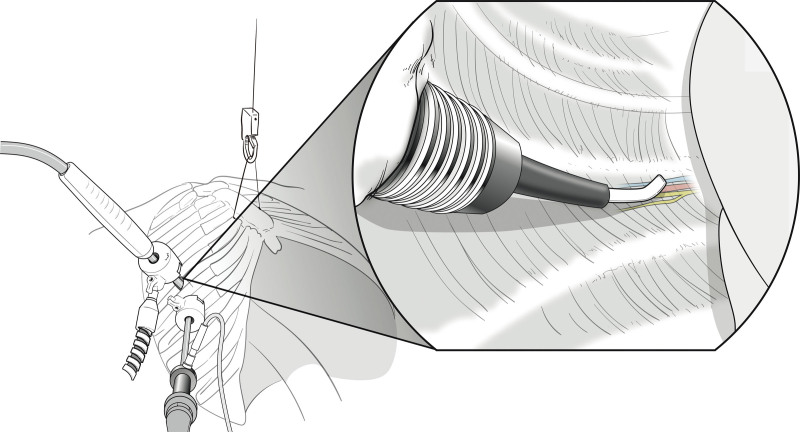
Scheme of thoracoscope-guided intraoperative cryoablation of intercostal nerve.

In the intraoperative cryoanalgesia group, following the skin incisions, a 12 mm port was inserted for cryoablation, and an additional 5 mm port was inserted for the thoracoscope. Insufflation of carbon dioxide gas was performed for a better exposure of the intercostal nerves. The cryoICE^TM^ probe was inserted into the ipsilateral thoracic cavity under thoracoscopic visualization. This procedure required approximately 2 minutes of ablation, with a temperature of -70°C at each of the targeted intercostal nerves. The targeted intercostal nerves were at the level of bar insertion, as well as one interspace above and below. Thereafter, MIRPE was performed (Fig. 1).

### 2.7. Epidural analgesia

In the epidural analgesia group, epidural patient-controlled anesthesia catheters were placed at the T5-6 level using a sterile technique before surgical procedure under general anesthesia. After placement, ropivacaine 0.15% and fentanyl 5 μg mL^-1^ were mixed and 10 mL of solution was given for bolus. An epidural patient-controlled anesthesia consisting of ropivacaine 0.15% and fentanyl 20 μg kg^-1^ and 0.9& saline in a total volume of 250 mL was provided for 48 hours after surgery at a basal rate of 5 mL hour^-1^, bolus dose of 0.5 mL, and lockout time of 15 minutes.

### 2.8. Postoperative pain management

The baseline strategy for pain management did not differ between the 2 groups. All patients received scheduled oral and nonsteroidal anti-inflammatory drugs. If patients were uncomfortable with pain, we added more oral narcotics. In addition to the baseline medications, intravenous opioids or fentanyl patches were administered as needed to encourage early ambulation. As pain improved, intravenous narcotics or patches were discontinued, and the pain was managed with oral medications alone. If a patient was under the age of 10 years, nonsteroidal anti-inflammatory drugs alone were administered.

### 2.9. Quantification of postoperative pain

It is usually difficult to quantify postoperative pain. Generally, patients are asked to rate the severity of their pain on a visual analog scale or a numeric pain intensity scale (NPIS). However, these are somewhat subjective. Therefore, we planned to use additional objective values, such as postoperative hospital stay and opioid dose consumed.

### 2.10. Statistical analyses

Continuous variables are expressed as medians and ranges, and categorical variables are expressed as counts and percentages. Continuous variables were compared using the Mann–Whitney test, and categorical variables were compared using Fisher exact test. The Bonferroni correction and Brunner and Langer method were used to compare the daily opioid requirement in both groups. Statistical analyses were conducted using SPSS (Version 25.0; SPSS Inc, Chicago) and SAS (version 9.4; SAS Inc, Cary, NC). Statistical significance was set at *P* < .05.

## 3. Results

Of the 64 patients, 51 (80%) were male, and the median age was 15 years (range, 4–33 years). The median MME consumed was 45 mg (range, 0–1360 mg), and the median postoperative hospital stay was 4 days (range, 2–6 days). The median duration of surgery was 70 minutes (range, 28–126 min), and the median Haller index was 4.02 (range, 2.76–13.23). Thirty-eight patients underwent cryoanalgesia, and the remaining 26 patients received epidural analgesia.

The baseline characteristics, such as age, sex, weight, height, and body mass index did not differ between the 2 groups (Table 1). Haller index was slightly larger in the epidural analgesia group; however, the *P* value was not statistically significant (*P* = .052). As expected, the median duration of surgery was longer in the cryoanalgesia group than in the epidural analgesia group (55 vs 84 min, *P* < .001).

**Table 1:** Comparison between the epidural analgesia and cryoanalgesia groups.

Variables	Epidural analgesia	Cryoanalgesia	*P* value
	(n = 26)	(n = 38)	
Age, y	14 (11–25)	17 (4–33	0.284
Sex, n (%)	21 (81%)	30 (79%)	0.859
Male	5 (19%)	8 (21%)	
Female			
Weight, kg	51 (40–78)	53 (17–79)	0.389
Height, cm	168 (155–185)	171 (109–189)	0.647
BMI, kg/m^2^	18 (14–23)	19 (13–23)	0.286
Haller’s index	4.44 (2.86–13.23)	3.85 (2.76–9.70)	0.052
Operation time, min	55 (28–91)	84 (47–126)	<.001
NPIS	5 (3– 7)	2 (1–4)	<.001
MME, mg	634 (227–1360)	19 (0–455)	<.001
POD, d	5 (3–6)	3 (2–4)	<.001

**Figure 2.**
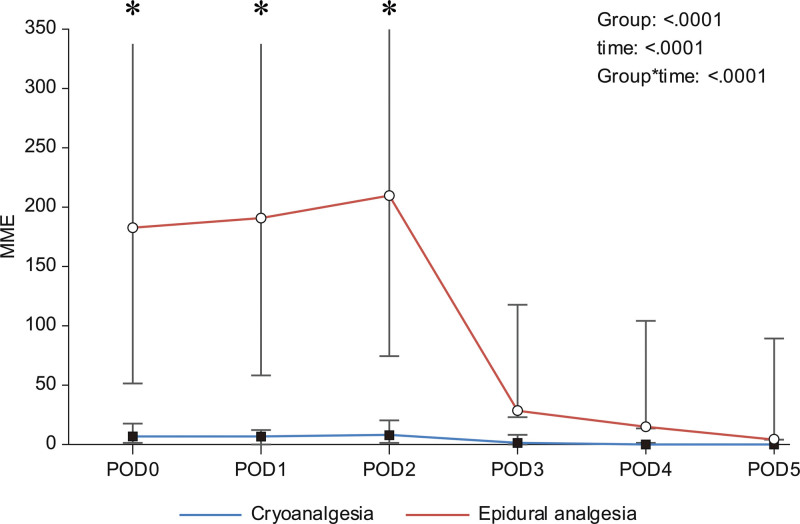
Comparison of the daily requirement of opioids between the cryoanalgesia and epidural analgesia groups.

Compared with the epidural analgesia group, the cryoanalgesia group had significantly lesser NPIS, lower MME and shorter postoperative hospital stay (5 vs 2; *P* < .001, 19 mg vs 634 mg, *P* < .001; 3 days vs 5 days, *P* < .001). No complications occurred intraoperatively or during the hospital stay. The daily NPIS and the requirement of opiates were significantly higher in the epidural analgesia group during the hospital stay (Table 2, Table 3, and Fig. 2).

**Table 2:** Comparison of daily NPIS between the epidural analgesia and cryoanalgesia groups.

POD	Epidural analgesia	Cryoablation	Overall
	(n = 26)	(n = 38)	*P* value
**0**	7 (4–9)	3 (1–3)	<.001
**1**	4 (1–7)	2 (0–4)	<.001
**2**	4 (1–9)	1 (0–4)	<.001
**3**	3 (1–7)	1 (0–3)	<.001
**4**	2 (1–7)	1 (0 –2)	<.001
**5**	2 (1–5)	0	<.001

**Table 3:** Comparison of daily MME required between the epidural analgesia and cryoanalgesia groups.

POD	Epidural analgesia	Cryoablation	Overall	Bonferroni post-hoc
	(n = 26)	(n = 38)	*P* value	*P* value
**0**	186 (0–396)	4 (0–109)	<.001	<.001
**1**	195 (76–397)	6 (0–122)	<.001	<.001
**2**	193 (76–420)	6 (0–140)	<.001	<.001
**3**	28 (0–105)	2 (0–92)	<.001	<.001
**4**	22 (0–102)	0 (0–88)	<.001	<.001
**5**	1 (0–89)	0	<.001	<.001

## 4. Discussion

Pain management following MIRPE is challenging. If pain is inadequately managed, postoperative ambulation might be compromised, potentially leading to several complications, including pneumonia, prolonged hospital stays, and higher doses of opioid agents. To date, several approaches have been adopted for better management of postoperative pain. These include epidural analgesia, intravenous patient-controlled analgesia, and subcutaneous infusion of local anesthetics. Among these, opioid agents have been widely used.^[6,11,12]^ However, opioids might not be the best option for managing pain in patients with pectus excavatum, as the majority of them are pediatric patients.

During cryoablation, gases from the cryoprobe and cryomachine undergo rapid expansion, and because of the Joule-Thomson effect, the temperature rapidly decreases. This sudden decrease in temperature induces axonotmesis, which refers to second-degree nerve injury. This process freezes the axons while leaving the fibrous neural structures (epineurium, perineurium, and endoneurium) intact. Due to the frozen axons, the transfer of electrical signals is prevented, and an analgesic effect is obtained. Subsequently, Wallerian degeneration progresses from the point of injury to the nerve endings. Intact fibrous neural structures allow axonal regeneration at the rate of approximately 1 to 3 mm/day. The continuous flow of blood acts as a heat sink to the intercostal vessels that are intact during cryoablation.^[13–16]^

Since the first cryoprobe was developed by Cooper in 1961,^[17]^ it has been used to manage pain in various surgeries, including craniofacial, chest wall, abdominal and pelvic, lower back, and lower extremity. In addition, cryosurgery is indispensable in dermatology.^[17–19]^ Nelson et al^[20]^ introduced cryoablation to manage post-thoracotomy pain, and subsequently, recent studies reported that cryoablation could be used for pain management after pectus excavatum correction.^[13,21–24]^ Cryoanalgesia has been reported to show promising results compared with other pain control modalities. Similar to our study, Keller et al^[9]^ and Graves et al^[21]^ also demonstrated that patients undergoing cryoablation of the intercostal nerves during MIRPE had a shorter length of hospital stay and lesser consumption of opioid agents than those treated with epidural analgesia.

Theoretically, 12 minutes was required to perform cryoablation in our protocol: 2 minutes per nerve for 3 nerves on each side. Our data showed that the duration of surgery was not statistically different between the 2 groups. Skin incisions were the same for the port and bar insertion; thus, it did not significantly increase the total duration of the procedure. Moreover, unlike in other studies, we performed cryoablation at 6 intercostal nerves, and not 10. In this study, we compared the daily narcotic requirements between the 2 groups. Compared with the cryoanalgesia group, MMEs from postoperative days 0 to 2 were significantly higher in the epidural analgesia group due to the infusion of opioids. However, daily MMEs were constantly low until discharge in the cryoanalgesia group. The differences between the groups decreased after the removal of the epidural infusion catheter. In our protocol, epidural catheters were removed when the epidural infusion was exhausted. We set the infusion for 48 to 72 hours. This led to a significant difference in MME consumption between the groups on postoperative days 0 to 2.

Neuralgia and numbness of the chest wall are 2 common concerns related to cryoanalgesia. Zobel et al^[25]^ reported that there was minimal risk of postcryoablation neuropathic pain in pediatric patients. In 25% of adult patients, the numbness did not resolve, and 3 patients had persistent neuropathic pain. In this study, we aimed to address these concerns. We hypothesized that the more the number of intercostal nerves blocked, higher would be the rate of complications. Therefore, we performed cryoablation at 6 intercostal levels instead of 10. Consequently, in the cryoanalgesia group in this study, 3 patients had no symptoms, whereas 7 had numbness. However, the degree of numbness improved over time in all 7 patients.

## 5. Limitations

This study had some limitations. First, the sample size was small. Second, the study design was retrospective in nature. Third, more information, such as longer follow-up duration, is necessary.

Despite these limitations, our study had several strengths. Cryoablation in this study was performed for fewer intercostal nerves compared with that in the previous studies, and our patients showed good recovery. The consumption of narcotics was significantly reduced, and the duration of hospital stay was shorter in the cryoablation group.

## 6. Conclusion

The results of this study showed that intraoperative cryoanalgesia could be easily performed to reduce the postoperative hospital stay and opioid dose. This illustrates that it could be an effective and safe modality for pain management following MIRPE.

## Acknowledgments

The authors thank Medical Illustration & Design, part of the Medical Research Support Services of Yonsei University College of Medicine, for all artistic support related to this work.
